# The physiological basis and clinical significance of lung volume measurements

**DOI:** 10.1186/s40248-017-0084-5

**Published:** 2017-02-09

**Authors:** Mohamed Faisal Lutfi

**Affiliations:** Department of Physiology, Faculty of Medicine and Health Sciences, Al-Neelain University, Khartoum, Sudan

**Keywords:** Lung volumes, Lung capacities, Obstructive, Restrictive, Spirometry

## Abstract

From a physiological standpoint, the lung volumes are either dynamic or static. Both subclasses are measured at different degrees of inspiration or expiration; however, dynamic lung volumes are characteristically dependent on the rate of air flow. The static lung volumes/capacities are further subdivided into four standard volumes (tidal, inspiratory reserve, expiratory reserve, and residual volumes) and four standard capacities (inspiratory, functional residual, vital and total lung capacities). The dynamic lung volumes are mostly derived from vital capacity. While dynamic lung volumes are essential for diagnosis and follow up of obstructive lung diseases, static lung volumes are equally important for evaluation of obstructive as well as restrictive ventilatory defects. This review intends to update the reader with the physiological basis, clinical significance and interpretative approaches of the standard static lung volumes and capacities.

## Background

**Fig. 1:**
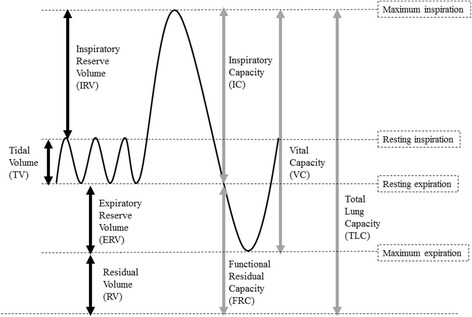
Standard lung volumes and capacities from a spirometer trace. The solid *black* and *gray* arrows indicate lung volumes and capacities respectively

Four standard lung volumes, namely, tidal (TV), inspiratory reserve (IRV), expiratory reserve (ERV), and residual volumes (RV) are described in the literature. Alternatively, the standard lung capacities are inspiratory (IC), functional residual (FRC), vital (VC) and total lung capacities (TLC). Figure 1 gives a schematic summary of the standard lung volumes and capacities [1–3]. RV constitutes part of FRC as well as TLC and, therefore, these capacities are impossible to measure through simple spirometers. The procedures used for measurement of RV, FRC and TLC are based on radiological, plethysmographic or dilutional techniques (helium dilution and nitrogen washout methods) [4]. However, body plethysmography and dilutional techniques may under-and overestimate lung volumes and capacities, respectively [5]. For the details of the procedures, advantages, disadvantages and recommendations for best practice of these techniques, the reader can refer to the reports revised and published by the joint committee of ATS/ERS [6].

The way how static lung volumes and capacities change in different physiological/pathological conditions depends on the understanding of the mechanics of breathing and the physiological determinants of pulmonary ventilation, which will be discussed in the following paragraphs.

## Mechanics of breathing

Towards the end of tidal expiration, the lungs tend to recoil inward while the chest wall tends to recoil outwards. These two opposing forces lead to a negative pressure within the potential space between the parietal and visceral pleurae. The negative intrapleural pressure (P_Pl_) is one of the important factors that keep the patency of small airways, which lack cartilaginous support. The rhythmic contraction of inspiratory muscles causes cyclic changes in the dimensions of the thoracic cage and consequently comparable cyclic fluctuation of P_Pl_.

**Fig. 2:**
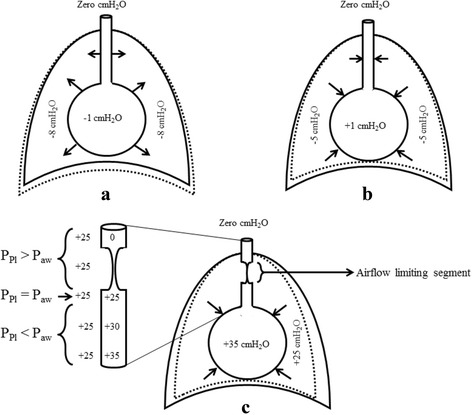
Intrapleural and alveolar pressures towards the end of inspiration (**a**), expiration (**b**), and forceful expiration (**c**). The dotted line indicates the change in thoracic dimensions during **a**, **b** and **c** compared with the previous phase of the respiratory cycle

During tidal inspiration, P_Pl_ drops from −5 to −8 cmH_2_O enforcing the intra-alveolar pressure (P_alv_) to drop one cmH_2_O below atmospheric pressure (P_atm_), Fig. 2a. As a result, air flows into the alveoli. The drop of P_Pl_ also decreases the airways resistance by dilating the small airways and thus enhancing the air flow further. The sequence of events reverses during tidal expiration. When inspiratory muscles relax, dimensions of the thoracic cage decrease, P_Pl_ increases from −8 back to −5 cmH_2_O and P_alv_ increases one cmH_2_O above P_atm_. As a result, air flows outside the alveoli following the pressure gradient, Fig. 2b. Tidal expiration is therefore a passive process, which needs no further muscle contraction. During tidal breathing, whether inspiratory or expiratory, intra-airways (P_aw_) pressure is always more than P_Pl_. This explains why small airways are always opened, even at the end of tidal expiration.

**Fig. 3:**
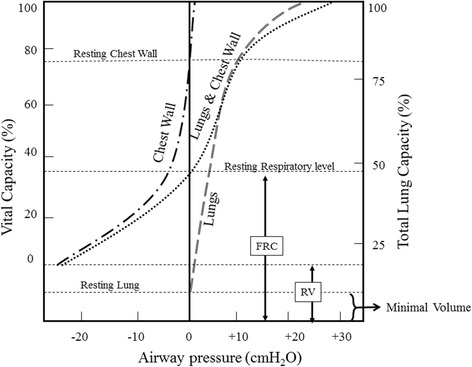
Static PVC of the lungs and chest wall. The lung and chest wall curve was plotted by the addition of the individual lung and chest wall curves

If inspiration above the tidal limit is required, accessory muscles of inspiration must be activated. Thoracic cage expands more leading to higher drop in P_Pl_ and P_alv_ compared with tidal inspiration, which explains why more air is delivered to the alveoli compared with tidal inspiration. Alternatively, expiration below the tidal level is an active process that requires contraction of expiratory muscles. During forceful expiration, the thoracic cage is compressed to the maximum. Both P_Pl_ and P_alv_ rise above P_atm_; however, P_alv_ remains more than P_Pl_ due to the effect of elastic recoil pressure (P_el_) of the alveolar wall. As demonstrated in Fig. 3c, P_aw_ decreases from the area next to the alveoli upwards. This gradual drop in P_aw_ is secondary to simultaneous increase in the airways resistance towards the trachea. Taking into consideration the relatively constant P_Pl_ around the lung, each small airway can be subdivided into three segments (Fig. 2c):

- An inflated segment, where P_Pl_ is lower than P_aw_.
- An equal pressure point, where P_Pl_ is equal to P_aw_.
- An airflow limiting segment, where P_Pl_ is higher than P_aw_.

Development of airflow limiting segments occurs in small airways that lack cartilaginous support and explains why the lungs cannot be empty completely. What limits airflow upon forceful expiration was previously explained by development of choke points i.e. the points where local flow velocity equalizes the local speed of pressure wave propagation (wave speed theory) [7, 8]. This is akin to a waterfall in which height and flow upstream the river are unlikely to affect the speed of the free falling water; nevertheless, if waterfall is broader, an extra water will be displaced. It is important to note that upon forced expiration, the increase in P_alv_ is accompanied by gas compression within the lung. This will result in reduction of both lung volume and P_el_. The decrease in P_el_ in turn attenuates the driving as well as the distending pressures at the choke points. This explains why the actual volume of forcefully expired air is always less than that measured with body plethysmograph. Based on the preceding narrative, it is easy to interpret why FEV_1_ measured with spirometer (FEV_1-Sp_) is typically less than that measured with body plethysmograph (FEV_1-Pl_) by an amount equal to thoracic gas compression volume (TGCV) [9–11].

Expiration after development of airflow limiting segments is effort independent. What remains in the lungs when small airways start to close is called the closing capacity (CC) [12, 13]. Alternatively, RV remains in the lung when all small airways are closed. The volume of air expired between CC and RV is called the closing volume (CV).

It is evident from the above description that pulmonary ventilation depends on the airways resistance offered to the airflow and expansibility (compliance) of the lungs and the thoracic cage. These two major determinants of pulmonary ventilation are crucial for understanding the pattern of change in static lung volume in different types of lung diseases.

1. Airways resistance

The tracheobronchial tree undergoes successive dichotomizations, where the airways become narrower but more distensible as we proceed downward. It is, therefore, difficult to apply simple laws of physics that govern fluid flow across single, non-branched, non-distensible tube system to evaluate respiratory airways resistance. For example, the lowest airways resistance resides on smallest bronchioles but not large airways. Because bronchioles are arranged in parallel, their resistances depend on the total cross sectional area of all bronchioles rather than the radius of a single bronchiole.

Airways resistance is inversely proportional to the lung volume. P_Pl_ decreases significantly upon inspiration, which enhances distension of airways especially small bronchioles. At higher lung volumes, attachments from the alveolar walls pull small airways apart and hence enhance the effect of P_Pl_ on decreasing airways resistance. In contrast, airways resistance increases significantly during forceful expiration due to formation of flow limiting segments.

2. Compliance of the lung and the chest wall

Compliance is a physical term used to predict the change in volume per unit change in the transmural pressure (P_T_) i.e. the pressure difference across two sides of a wall. From physiological perspective, the P_T_ for the lungs (trans-pulmonary pressure), chest wall (trans-chest wall pressure) and respiratory system (trans-respiratory pressure) are calculated by subtracting P_alv_ – P_Pl_, P_Pl_ – P_atm_ and P_alv_ – P_atm_, respectively. According to physics, if P_T_ is equal to zero then the system is resting i.e. neither inflating nor deflating.

Like lung volumes, the lung compliance can be measured under static and dynamic conditions. Figure 3 shows the static pressure volume curves (PVC) of the lungs and the chest wall. The entire lung PVC in Fig. 3 falls within the positive limb of P_aw_, suggesting the tendency of the lungs to collapse at any degree of pulmonary inflation. The lungs are never rested within the chest cage i.e. trans-pulmonary pressure never reaches zero. If removed outside the body then trans-pulmonary pressure can reach zero; however, the lung will not be empty completely, Fig. 3.

In comparison, chest wall tends to recoil outward as far as the lung is filled with 80% of TLC or less. At lung volumes more than 80% of TLC, the chest wall recoils inward, Fig. 3.

The lung–chest wall system is rested when P_alv_ is equal P_atm_ and the lungs are filled with FRC. At this point the inward recoil tendency of the lungs is equal to the outward recoil tendency of the chest wall, Fig. 3.

**Fig. 4:**
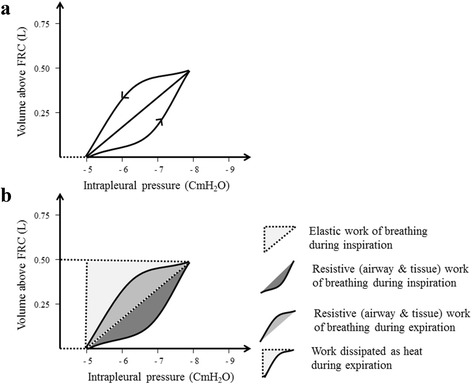
**a** Dynamic PVC of the lungs. **b** Work of breathing

The PVC of the lungs can also be recorded during breathing to evaluate dynamic lung compliance. It is evident from Fig. 4a that dynamic PVC for inspiration and expiration are separate and do not follow the same pathway. This phenomenon is known as hysteresis and can be explained by the variations of surface tension at alveolar air-fluid interface during inspiration and expiration. Pulmonary surfactant is a natural substance that reduces surface tension of the fluid layer lining the alveoli. During inspiration, alveolar surface tension is likely to increase because pulmonary surfactant spreads over a wider alveolar surface. The reverse occurs during expiration, where pulmonary surfactant condenses in a smaller alveolar surface. Hysteresis can also be explained by progressive opening “recruitment” and closure “derecruitment” of small airways and alveoli during inspiration and expiration respectively.

The work of breathing is usually estimated by the area under the dynamic PVC of the lungs (Fig. 4b). During inspiration, the work needed to overcome elastic forces of the chest wall, lungs parenchyma and alveolar surface tension is called elastic work of breathing. In addition, a resistive work is needed during inspiration to overcome tissue and airways resistance. In contrast to inspiration, only resistive work of breathing is required during expiration. Under physiological condition the work needed for inspiration is more than that needed for expiration. The energy stored in the elastic lung structures during inspiration is partly consumed as expiratory resistive work and partly dissipated as heat (Fig. 4b).

**Fig. 5:**
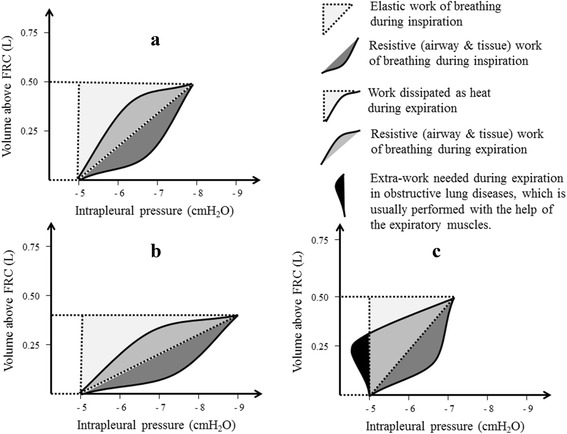
Work of breathing in  normal subjects (**a**) and patients with RLD (**b**) and OLD (**c**)

Physiologically, the diseases that affect the respiratory system are characterized by restrictive, obstructive or combined pattern of ventilatory defects [14, 15]. Restrictive lung diseases (RLD) are associated with decreased compliance of the lungs, chest wall or both. This results in rightward shift of static PVC of the lungs, chest wall or both [15]. It is evident from Fig. 5 (a and b) that decreased compliance of the lungs increases P_Pl_ needed for tidal inspiration, yet tidal volume is below the expected average. In RLD, the rightward shift of dynamic lung compliance curves increases the elastic work of breathing required for inspiration, which is usually compensated by rapid shallow breathing [16]. Causes of RLD may be intrinsic or extrinsic to the lung parenchyma. Examples of intrinsic causes are interstitial lung diseases, pneumonia and surfactant deficiency e.g. acute respiratory distress syndrome. Alternatively, respiratory muscles weakness, chest deformities, cardiomegaly, hemothorax, pneumothorax, empyema, pleural effusion or thickening are examples of extrinsic causes.

In obstructive lung diseases (OLD), the pulmonary compliance is normal or increased especially if emphysematous lung changes co-exist. No extra-negative P_Pl_ is needed as dynamic lung compliance curves are either not displaced or shifted leftward if emphysematous lung changes developed (Fig. 5c). The main defect in OLD is increased airways resistance, especially during expiration. Normally, expiration is a passive process as the energy needed to overcome expiratory resistive work of breathing is stored in the elastic fibers of the lung during inspiration. It is evident from Fig. 5c that expiration is not completely passive if OLD exists as an extra-work is needed during expiration, which is usually performed with the aid of expiratory muscles. Famous examples of obstructive pulmonary diseases include bronchial asthma, emphysema, chronic bronchitis and bronchiectasis.

## Physiological determinant of the static lung volumes and capacities 

### Age

**Fig. 6:**
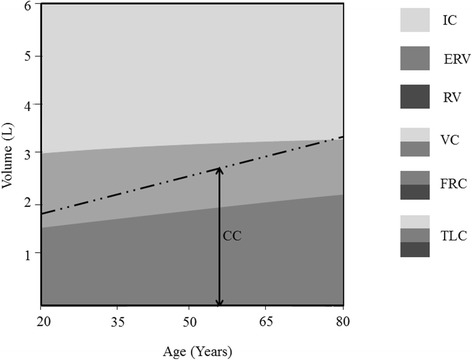
Changes in static lung volume and capacities with age

The lung volumes increase steadily from birth to adulthood. The lungs mature at the age of 20–25 years, yet only minimal changes occur in the lung volumes over the following 10 years [17]. After 35 years, aging is associated with gradual changes in the lung volumes and other pulmonary functions [18]. These changes include enhanced static lung compliance due to diminished alveolar elastic recoil and depressed chest wall compliance due to stiffening and increased outward recoil of the thoracic cage [19, 20]. As a result of these changes in the lung and chest wall compliances, the inward recoil of the lung balances the outward recoil of the chest at higher FRC as age progress [12, 13]. These variations in lung and chest wall compliances act synergistically to cause early closure of small airways upon forced expiration and hence explain increased RV in elder people [19]. As shown in Fig. 6, TLC corrected for age remains almost constant throughout life. However, gradual increase in FRC and RV with age results in simultaneous decrease in IC and VC, respectively [17]. It is also apparent from Fig. 6 that the increase in CC when age advances is more compared with FRC. This results in a reduction of the difference between these two capacities i.e. D (FRC ─ CC) as age progress. In the sitting position, CC is likely to exceed FRC at an age of 75 years or more [12] (Fig. 6), but much earlier in the supine position (≈44 years) [13].

### Gender

Standard morphometric methods confirmed that males had larger lung size, more respiratory bronchioles and wider airways diameters compared with females with the same age and stature [21, 22]. These anatomical lung differences between males and females explain the gender variations in static lung volumes and capacities. Males tend to have larger anthropometric measurements and are, therefore, more likely to have increased static lung volumes and capacities [23].

### Anthropometric measurements

Tall stature is typically associated with higher static lung volumes and capacities [24]. Increased body weight is associated with lower lung volumes in obese subjects [25]. Central obesity preferentially depresses chest wall compliance leading to marked decrease in FRC and ERV [26]. Waist-to-hip ratio could be a better predictor for fat distribution than BMI [27]. However, the effects of obesity on the highest (TLC) and lowest (RV) lung volumes are modest [28]. In athletes, repeated muscular exercise increases muscle mass and consequently body weight. In such condition, the static lung volumes and capacities are expected to increase with weight [29–32]. Increased total body fat content, therefore, seems better than high BMI as an indicator of obesity as well as predictor for decreased static lung volumes and capacities [33].

### Ethnicity

Previous studies demonstrated ethnic differences in the lung volumes/capacities [34, 35]. Such variations were largely attributed to anthropometric differences between different ethnic groups. For example, white Americans of European descent have larger trunk/leg ratio, and consequently higher lung volumes, compared with black Americans of African descent [36]. Other studies failed to justify ethnic differences in lung volumes by the variations in chest contours and suggest differences in inspiratory muscle strength and/or lung compliance as alternative explanation(s) [37]. Recently, GLI (Global Lung Initiative) offered spirometric prediction equations, that also considered ethnic differences, to be used worldwide [38].

### Other factors

Although age, gender, weight, height and ethnicity are the main physiological determinants of the static lung volumes/capacities, other factors should be considered while interpreting results of spirometry.

Lung volumes correlate well with the level of physical activity [39], regular exercise, especially swimming and endurance training [32]. Alternatively, ascending to high altitude may decrease lung volumes probably due to increased pulmonary blood flow, pulmonary edema or premature small airways closure [40]. Alterations in lung volumes associated with high altitude are usually temporal and resolve after returning to the sea level [41].

The position of the subject is important while measuring lung volumes and capacities [42]. Compared with the standing position, the effect of gravity on abdominal viscera is less at sitting position and least if lying supine [43]. The supine position, therefore, compromises diaphragmatic movement and chest wall recoil during breathing. FRC and ERV are higher upon standing compared with sitting and supine positions [44]. Increased intra-abdominal pressure during pregnancy also causes decreased FRC and ERV [45].

## Interpretation of static lung volumes and capacities

The quality and accuracy of the test(s) used for estimation of the lung volumes/capacities should be ensured before interpretation [46]. The measurement of the lung volumes is not an easy task and requires cooperative patients and qualified technicians. Personnel in the pulmonary laboratory must be able to judge precisely test acceptability and reproducibility criteria for the different techniques used for estimation of the lung volumes/capacities [46]. Special attention should be given to the accuracy of the method used for estimation of the static lung volumes and capacities. Plethysmography was claimed to overestimate while dilutional techniques may underestimate the true measurements of the lung volumes and capacities [5].

The normal lung volumes and capacities can be predicted based on gender, age, weight, height and ethnicity of the subject [47]. Although authorized spirometric reference values are available for most populations, normal ranges of lung volumes and capacities were not established in others yet. Static lung volumes and capacities are frequently expressed as a percent of the predicted value, where 80% and 120% are considered as the lower (LLN) and upper (ULN) limits of normal. However, the use of these cut-off points may be misleading in characterizing ventilatory defects in some pulmonary diseases if only simple spirometry is performed [48, 49].

## Patterns of changes of static lung volumes and capacities in pulmonary diseases

### Restrictive lung diseases

**Fig. 7:**
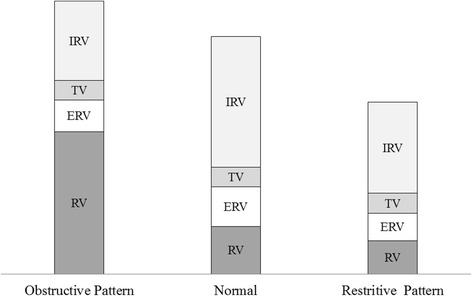
Typical changes in the static lung volumes and capacities in RLD and OLD

Diseases associated with diminished pulmonary compliance interfere with lung expansion and ultimately reduce static lung volumes/capacities, Fig. 7. According to ATS/ERS, restrictive ventilatory defect is ideally confirmed by a reduction in TLC below the 5^th^ percentile of the predicted value, and a normal FEV_1_/VC [46], though most pulmonary laboratories use VC instead because it constitutes most of the TLC [50, 51]. The use of VC as a surrogate for TLC in diagnosis of RLD assumes a proportional decrease in RV and TLC so that their ratio remains constant [46, 52]. Simultaneous increase of RV with VC reduction is indicative of obstructive lung disease because of small airway closure or expiratory flow limitation [53]. Therefore, decreased VC readings are better interpreted in conjunction with other clinical and spirometric indicators of OLD, especially if measurements of RV and TLC are not available [54]. According to Aaron et al., the chances of restrictive ventilatory defect are 2.4% and 58% in those with normal and low VC readings, respectively [55]. These findings suggest that normal VC may be effective in exclusion, but not confirmation, of RLD. This hypothesis is further supported by Vandevoorde et al., who concluded that RLD can be ruled out if FVC is more than 100% of predicted in males or greater than 85% of predicted in females [56].

If thoracic cage expansion is restricted, rightward displacement of the chest wall static PVC takes place. This readjusts the point where the inward recoil of the lung equalizes the outward recoil of the chest wall at a lower FRC level. In cases with severe central obesity, decreased chest wall compliance reduces FRC and ERV [57]. According to Jones et al., FRC and ERV at a body mass index (BMI) of 30 kg/m^2^ were about 75% and 47% of the respective measurements for subjects with BMI of 20 kg/m^2^ [26]. The same study failed to demonstrate a significant effect of high BMI on RV/TLC ratio, which indicates proportional reduction in RV and TLC in overweight and obese subjects. Marked reduction of FRC and ERV in such cases may induce premature formation of flow limiting segments during quiet breathing, especially in the lower regions of the lungs [57]. This implication is further supported by the studies that confirm an inverse relationship between FRC and airway resistance in obese patients [58, 59]. Furthermore, temporal variability of ventilation heterogeneities increases in obesity when FRC falls approximately below 65% of predicted or ERV below 0.6 l, promoting ventilation perfusion inhomogeneity and eventually hypoxemia [60].

### Obstructive lung diseases

ATS/ERS defined obstructive ventilatory defect as “disproportionate reduction of maximal airflow from the lung in relation to the maximal volume (i.e. VC) that can be displaced from the lung” [46]. Obstructive ventilatory defect is ideally confirmed by FEV_1_/VC ratio below the 5^th^ percentile of the predicted value [46].

VC can be measured while doing slow (SIVC) or forceful (FIVC) inspiration starting from RV up to the level of TLC [61, 62]. Likewise, VC can be estimated while doing slow (SEVC) or forceful (FEVC) expiration starting from TLC up to the level of RV [62, 63]. Taking into consideration the variations in airways resistance between inspiration and expiration, it is easy to conclude that different types of VC are not equal. The differences between the four types of VC are minimal in those with no ventilatory defect [61]. In patients with OLD, FIVC > SIVC > SEVC > FEVC [50, 63]. FEVC (commonly abbreviated as FVC) is, therefore, the most affected type of VC in cases with severe obstructive lung disease [63].

In OLD, formation of flow limiting segments occurs early due to narrowing of airways. Premature closure of small airways in OLD results in increased RV. In such conditions, RV may increase at the expense of VC so that TLC remains unchanged [53]. Alternatively, RV may increase while VC remains almost unchanged leading to higher TLC values [64]. In both scenarios, RV/TLC ratio is likely to increase irrespective of the changes in the VC, a fact that explains the superiority RV/TLC over TLC in evaluation of OLD [65].

Similar to the RV and VC changes occurring in patients with OLD, FRC may increase at the expense of IC so that TLC remains unchanged [4]. IC can directly be measured by spirometry, which is advantageous in places where there are no facilities to measure RV and TLC. There are accumulating evidences that indices derived from IC are helpful to assess severity, prognosis and response to treatment of many OLD [66–69]. According to Yetkin and Gunen, IC is more efficient than FEV_1_ is assessing severity of COPD during acute exacerbation [68]. In another study, COPD patients with IC/TLC ratio < 25% are more likely to have unscheduled doctor visits due to exacerbations or need of carefully monitored treatment [69]. This fact is further supported by the finding of French et al., where IC/TLC ≤ 25% was identified as significant predictor of death in patients with emphysematous COPD [67].

It is evident from the above reports that air trapping in obstructive ventilatory defects correlates positively with RV, FRC, TLC and RV/TLC, but negatively with VC, IC and IC/TLC. As described earlier, FIVC > SIVC > SEVC > FEVC in patients with OLD [50, 63]. Accordingly, lung hyperinflation can also be evaluated by assessing the difference between FIVC and FEVC [62, 63, 70, 71]. Larger difference between FIVC and FEVC had been validated not only as an efficient index of severity of airflow limitation, but also as powerful predictor of exercise tolerance in patients with COPD [62, 71]. Likewise, lung hyperinflation secondary to air trapping can be estimated by calculating the difference between lung volumes measured by plethysmography and dilutional techniques. This assumption was validated by Tantucci et al. when they evaluated FRC in asthmatic patient by plethysmography (FRC_pl_) and helium dilution method (FRC_He_) following methacholine challenge test [72]. The results confirmed that comparing FRC_pl_ with FRC_He_ was helpful in identifying asthmatic patients at risk of tidal airway closure induced by methacholine. In addition, Tantucci et al. demonstrated significant correlation between (FRC_pl_ ─ FRC_He_) and the unventilated lung volume following provocation of bronchoconstriction [72]. Typical changes in the static lung volumes and capacities in OLD are summarized in Fig. 7.

It is important to note that FEV_1_ should be interpreted with caution when measured with spirometers (FEV_1-Sp_) rather than plethysmography (FEV_1-Pl_). As explaineed earlier, FEV_1-Sp_, but not FEV_1-Pl_, is biased by TGCV [9–11]. In a recent study involving asthmatic patients during methacholine challenge, FEV_1-Sp_ overestimated bronchoconstrictor response in those with larger lung volume [73]. FEV_1-Sp_ also overestimated bronchodilator response following administration of salbutamol to the same patients. In another study, FEV_1-Sp_ and FEV_1-Pl_ were simultaneously measured in 47 and 51 subjects with dominant emphysema and dominant chronic bronchitis, respectively [74]. The results confirmed larger lung volumes and lower FEV_1-Sp_ in emphysematous patients compared with those with dominant chronic bronchitis. When FEV_1-Pl_ was used instead of FEV_1-Sp_, the disease severity was less in classes with dominant emphysema than those with dominant chronic bronchitis. The study concluded that FEV_1-Sp_ was biased by TGCV more in patients with dominant emphysema because their TLCs were larger.

### Mixed obstructive and restrictive lung diseases

Decreased TLC in patients with spirometric evidence of airways obstruction e.g. RV above ULN or FEV_1_% below LLN is suggestive of mixed obstructive-restrictive lung diseases (MORLD). In MORLD, premature formation of flow limiting segments and diminished pulmonary compliance synergistically decrease FVC. The reduction in FVC sometimes exceeds that occurs in FEV_1_ and consequently results in relatively higher FEV_1_% [75]. This fact explains the findings of Balfe et al. study, which compared grading of airway obstruction based FEV_1_ (American Thoracic Society (ATS) recommendation) and FEV_1_% (Intermountain Thoracic Society (ITS) recommendation). According to Balfe et al. results, ATS recommendation graded 90% of 147 MORLD patients as having severe obstruction while ITS recommendation graded only 3% with the same degree of obstruction [76]. An additional evidence was given by another study that demonstrated an inverse correlation between FEV_1_% and RV/TLC in patients with MORLD [77]. Accordingly, adjustment of FEV_1_% for the reduction in TLC is likely to improve grading of the severity of obstruction in patients with MORLD. This assumption was verified in a study evaluating 199 patients with MORLD, where FEV_1_%/TLC was used for adjustment for the degree of restriction [78]. Based on ATS/ERS grading, 76% and 11% of MORLD patients were classified as having severe and mild-to-moderate obstruction, respectively. In comparison, the adjusted FEV_1_% (FEV_1_%/TLC) classified 33% and 44% of the same patients as having severe and mild-to-moderate obstruction. The study concluded that subdividing FEV_1_% by TLC resulted in an appropriate severity classification of obstruction when restriction coexists [78].

## Non-specific pattern of changes in lung volumes and capacities

The term non-specific pattern (NSP) is used to describe coexistence of low FEV_1_ and FVC with normal TLC and FEV_1_% [46, 79]. Although lower values of both FEV_1_ and FVC are associated with obstructive as well as restrictive ventilatory defect, the other components of NSP (i.e. normal TLC and FEV_1_%) minimize the possibility of these conditions. Hypothetically, if RV increases while TLC remains unchanged, VC and consequently FEV_1_ are expected to decrease below the normal limits. NSP may, therefore, reflect an obstructive impairment of small airways, where RV expands at the expense of VC so that TLC remains unaffected [53, 79]. However, NSP was also demonstrated in patients with restrictive ventilatory defects [80]. In a previous study, in depth evaluation of a random sample of patients with the NSP confirmed OLD and RLD as a possible cause in 68% and 32% of the examined subjects, respectively [80]. In another study, NSP persisted in 64% of 1,284 patients after 3 years follow up. Nonetheless, the NSP changed to RLD, OLD, MORLD and normal patterns in 16%, 15%, 2% and 3% of the studied patients, respectively [81]. Possible explanation for NSP in patients with restrictive ventilatory defects remains for further investigations and researches.

## Conclusions

Physiological factors that influence lung volumes/capacities include age, gender, weight, height and ethnicity, physical activity, altitude and others, which should be considered while interpreting results of spirometry. Likewise, the quality and accuracy of the test(s) used for estimation of the lung volumes/capacities should be considered before interpretation.

RLDs are ideally confirmed by low TLC, though most pulmonary laboratories use VC instead. VC instead of TLC may be effective in exclusion, but not confirmation, of RLD. Simultaneous increase in RV with VC reduction is indicative of obstructive lung disease. Therefore, decreased VC readings are better interpreted in conjunction with other clinical and spirometric indicators of OLD. In RLD like central obesity, decreased chest wall compliance reduces FRC and ERV, which may induce premature formation of flow limiting segments during quiet breathing.

Premature closure of small airways in OLDs results in increased RV. In such conditions, RV may increase at the expense of VC so that TLC remains unchanged. Alternatively, RV may increase while VC remains almost unchanged leading to higher TLC values. In both scenarios, RV/TLC ratio is likely to increase irrespective of the changes in the VC, a fact that explains the superiority RV/TLC over TLC in evaluation of OLD. Similarly, FRC may increase at the expense of IC so that TLC remains unchanged.

Decreased TLC in patients with spirometric evidence of airways obstruction is suggestive of MORLD. In such conditions, the reduction in FVC exceeds that occurs in FEV_1_ and consequently results in relatively higher FEV_1_%. The term NSP is used to describe coexistence of low FEV_1_ and FVC with normal TLC and FEV_1_%. NSP may reflect an obstructive impairment of small airways, where RV expands at the expense of VC so that TLC remains unaffected. NSP was also demonstrated in patients with restrictive ventilatory defects, which needs further investigations and researches.

## Abbreviations

ATS: American Thoracic Society
COPD: Chronic obstructive pulmonary disease
ERS: European Respiratory Society
ERV: Expiratory reserve volume
FEV_1_: Forced expiratory volume in the first second
FEV_1-Sp_: FEV_1_ measured with spirometer
FEV_1-Pl_: FEV_1_ measured with plethysmography
FEVC: Forced expiratory vital capacity
FIVC: Forced inspiratory vital capacity
FRC: Functional residual capacity
FVC: Forced vital capacity
IC: Inspiratory capacity
IRV: Inspiratory reserve volume
ITS: Intermountain Thoracic Society
LLN: Lower limits of normal
NSP: Non-specific pattern
OLD: Obstructive lung diseases
P_alv_: Intra-alveolar pressure
P_atm_: Atmospheric pressure
P_aw_: Intra-airways pressure
P_el_: Elastic recoil pressure
P_Pl_: Intrapleural pressure
P_T_: Transmural pressure
PVC: Pressure volume curves
RLD: Restrictive lung diseases
RV: Residual volume
SEVC: Slow expiratory vital capacity
SIVC: Slow inspiratory vital capacity
TGCV: Thoracic gas compression volume
TLC: Total lung capacity
TV: Tidal volume
ULN: Upper limits of normal
VC: Vital capacity
